# Melatonin: A Review of Its Potential Functions and Effects on Dental Diseases

**DOI:** 10.3390/ijms18040865

**Published:** 2017-04-19

**Authors:** Maria Permuy, Mónica López-Peña, Antonio González-Cantalapiedra, Fernando Muñoz

**Affiliations:** Anatomy, Animal Production and Veterinary Clinical Sciences Department, Veterinary Faculty, Universidade de Santiago de Compostela, Campus Universitario s/n, 27002 Lugo, Spain; monica.lopez@usc.es (M.L.-P.); antonio.cantalapiedra@usc.es (A.G.-C.); fernandom.munoz@usc.es (F.M.)

**Keywords:** melatonin, oral cavity, implants, periodontal disease

## Abstract

Melatonin is a hormone synthesised and secreted by the pineal gland and other organs. Its secretion, controlled by an endogenous circadian cycle, has been proven to exert immunological, anti-oxidant, and anti-inflammatory effects that can be beneficial in the treatment of certain dental diseases. This article is aimed at carrying out a review of the literature published about the use of melatonin in the dental field and summarising its potential effects. In this review article, an extensive search in different databases of scientific journals was performed with the objective of summarising all of the information published on melatonin use in dental diseases, focussing on periodontal diseases and dental implantology. Melatonin released in a natural way into the saliva, or added as an external treatment, may have important implications for dental disorders, such as periodontal disease, as well as in the osseointegration of dental implants, due to its anti-inflammatory and osseoconductive effects. Melatonin has demonstrated to have beneficial effects on dental pathologies, although further research is needed to understand the exact mechanisms of this molecule.

## 1. Introduction

Melatonin (*N*-acetyl-5-methoxy-tryptamine) is an indoleamine synthesised and secreted by the pineal gland and other organs, such as the retina, bone marrow, and intestines in a circadian pattern. Extrapineal sites contribute poorly, or only upon specific stimuli to circulating melatonin [1]. Melatonin influences numerous physiological actions that may be mediated by the binding of the indoleamine to membrane receptors in all tissues [2,3]. Due to its excellent lipophilic properties, melatonin is able to enter the subcellular compartment, finding it in high concentrations in the nucleus and the mitochondria of cells [4,5], being capable to bind to some cytosolic proteins like kinase-C [6], calmodulin [7] and calreticulin [8]. Currently, melatonin is not considered a hormone in the classical sense of the term, because it is synthesised in several organs and does not exert effects on a specific target [9], but it is rather a powerful cell protector against molecular damage.

**Figure 1:**
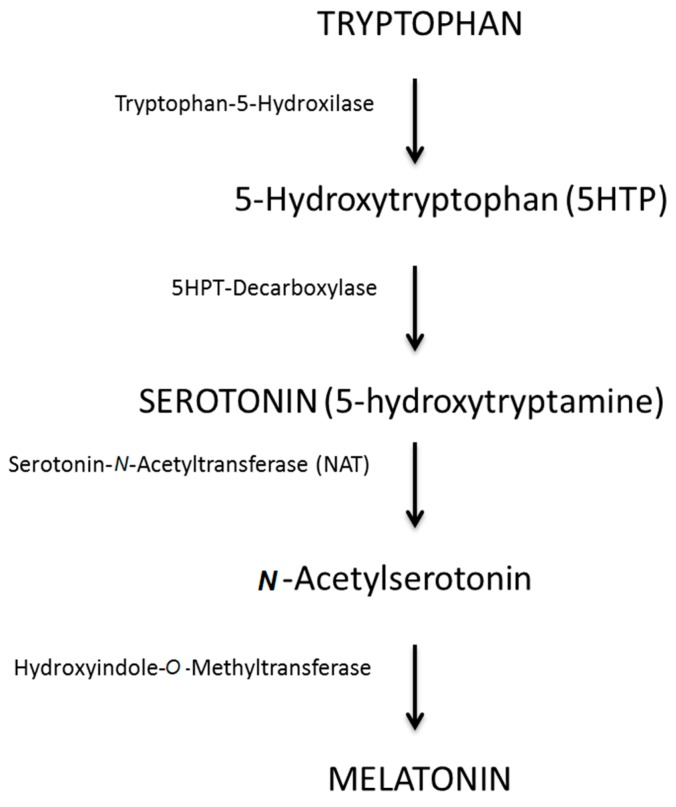
Melatonin synthesis and representation of its molecule (elaborated by the authors).

For the synthesis of melatonin, the pineal cells take up tryptophan from the blood and, through a hydroxylation and a decarboxylation process, turn it into serotonin. Serotonin is then converted into *N*-acetylserotonin through *N*-acetyltransferase and subsequently methylated to the final form of melatonin by the enzyme hydroxyindole-*O*-methyltransferase [10,11] (Figure 1). In healthy individuals, the maximum secretion of melatonin occurs between midnight and 2 a.m., then it decreases to a minimum during the day [12]. The production of melatonin declines after the age of 40–45 years, with a continuous reduction as age increases [11].

Seventy percent of the melatonin in blood is bound to proteins and only the free percentage (30% of the total melatonin) is able to diffuse into the surrounding tissues [1], including the oral cavity through saliva; thus, the proportion of plasma melatonin entering the oral cavity via the salivary glands ranges from 24% to 33%, appears to be stable [13] and there are no morpho-physiological barriers to melatonin; indole rapidly crosses the blood-brain barrier, the placenta, and all of the other potential cellular impediments [14]. The evaluation of the melatonin level in saliva is a reliable technique to monitor circadian rhythms and melatonin secretion.

Melatonin has numerous physiological functions in different parts of the body, such as the control of circadian rhythms [15,16], regulation of body temperature [17], regulation of sexual development and the reproductive cycle, especially in seasonal animals [18,19], and activation of the immune system [20,21]. In the oral cavity, melatonin has been recognised as an important substance with paracrine effects on nearby cells [22]; it also acts as an antioxidant and an anti-inflammatory and plays an important role in bone formation and in the reduction of bone resorption.

Melatonin can be administered by several routes (e.g., orally, intraperitoneally, directly in the effect-site in dental implants) and it is available as a supplement or as a prescription drug, depending on the country. This molecule has a long shelf life, is not expensive, has few side effects compared to other drugs, and can be used with a very wide safety margin. These characteristics, along with the wide range of effects on the tissues, make it a potential therapy for different purposes.

The aim of this review is to summarise the potential actions of melatonin in the oral cavity, focussing on implantology and periodontal disease.

## 2. Material and Methods

The PubMed database of the U.S. National Library of Medicine was used as the main electronic database in this review for the collection of data, performing a systematic search of the articles published in the literature on the subject. A commercially available software program (Endnote, Thomson Reuters, London, UK) was used for electronic title management.

The combination of keywords used for the electronic search were:

- melatonin
- melatonin and oral cavity
- melatonin and implant
- melatonin and periodontal disease
- melatonin and cancer
- melatonin and microorganisms, bacteria or virus

The obtained results were carefully evaluated and the most important findings related to the use and effects of melatonin on oral diseases are summarised below.

For a better understanding of the results, the present review was divided into the following sections:

1. Main effects of melatonin related to the oral cavity;
2. Melatonin and dental implants;
3. Melatonin and periodontal disease;
4. Other effects of melatonin on the oral cavity.

## 3. Results

### 3.1. Main Effects of Melatonin Related to the Oral Cavity

The role of melatonin in the oral cavity (both in physiological and pathological processes) is basically related to its antioxidant and anti-inflammatory effects, as well as acting as a mediator in bone formation and resorption [23].

Free radicals are molecules with an unpaired electron in their valence shell. Due to this electron-deficient state, they are highly reactive, causing damage to the adjacent molecules by abstracting an electron or donating it to them. Out of all the free radicals, those derived from oxygen or nitrogen could be highly destructive; given that most of the cells are of an aerobic nature, they generate reactive oxygen or nitrogen species (ROS and RNS), the antioxidant system mediated by enzymes or small molecules that can scavenge those free-radicals being particularly important under physiological conditions [24]. Melatonin was proven to directly neutralise ROS [25,26] acting as an antioxidant in several conditions and tissues.

Due to its antioxidant properties and its ability to detoxify free radicals, melatonin can also interfere in the bone resorption process, mediated by the osteoclasts acting at the level of the osteoclast lacuna and blocking the reactive oxygen species produced by the superoxide dismutase [27]. Another effect of melatonin in bone resorption is mediated through the downregulation of the receptor activator of nuclear factor κ-B ligand (RANKL)-mediated osteoclast formation and activation, which increases bone mass. This effect was achieved with pharmacological doses of melatonin [28].

With regard to bone formation, melatonin promotes osteoblast differentiation [29,30,31] and stimulates the formation of a new mineralised matrix [29,32]. It was observed that, in human osteoblasts in vitro, melatonin has the ability to stimulate, at micromolar concentrations, the proliferation and synthesis of collagen type I, other bone matrix proteins and bone markers (including alkaline phosphatase, osteopontin, and osteocalcin). It also reduces the osteoblast differentiation period from the usual 21 days to 12 [29,32]. Another study has shown that melatonin influences precursors of bone cells in the bone marrow of rats [33] and protects the bone cells from oxidative attacks [34].

Melatonin also has other effects on bone, different from those of formation and resorption, such as acting as a significant modulator of calcium metabolism, preventing osteoporosis and hypocalcaemia [35].

Another role of melatonin, important for bone regeneration, is that of mediator of angiogenesis [36]. Growth factors, such as the vascular endothelial growth factor (VEGF), are considered potential angiogenic modulators. Melatonin increases VEGF expression by exerting a significant pro-angiogenic activity in tissues, including bone [37]. In another study, melatonin also showed a positive effect on monocytes, cytokines, and fibroblasts having an impact on angiogenesis, as well [38].

### 3.2. Melatonin and Dental Implants

Missing and defective teeth are common in humans, especially in middle-aged and elderly people, as a consequence of aging. Due to this situation, a higher number of implants are needed for dental reposition. Improving implant success rate and achieving a faster osseointegration are important goals in dentistry. The osseointegration of implants is related to the direct apposition of new bone in contact with the implant surface [39], as well as to the remodelling of the pre-existing one. The production of new bone involves a cascade of different events, such as cell maturation and apposition, vascular invasion, and bone formation and mineralisation. The early stability of implants is extremely important to avoid their failure, and the process of integration may be accelerated through the local delivery of growth factors, as seems to be the case with the application of melatonin [2].

The use of melatonin to promote bone regeneration in dental implant placement was assessed in several studies, based on different animal models in which the authors employed melatonin alone or in combination with other substances, such as, growth hormone or porcine bone [2,40,41,42,43,44,45,46,47]. The studies using melatonin to promote dental implant stability evaluated in this review are summarised in Table 1.

The histomorphometric parameters used to evaluate the success of implant integration in the different studies were: bone to implant contact (BIC, which refers to the total length or percentage of the implant surface in contact with bone), bone density (BV/TV, which is the ratio of bone with respect to the total volume of the tissue), and new bone area and inter-thread bone (which is the bone inside the threads of the implant). In addition, several studies reported histological findings, such as the appearance of new tissue and its mineralisation, the different types of cells observed in the tissue and blood vessels formation.

Most studies evaluated in this review reported that in a short period of time after the implant placement, ranging between two and eight weeks, melatonin significantly increased BIC, BV/TV, new bone area, and inter-thread bone, leading also to an increase in the osteoblast proliferation in the peri-implant zone [2,42]. In a study using melatonin in combination with porcine bone in titanium implants, this combination significantly improved BIC, bone density, and new bone area in comparison with the use of porcine bone alone [41]. In a study conducted by Muñoz et al. [43] the use of melatonin, along with growth hormone in dogs, produced a significant improvement of all the osseointegration parameters at two and five weeks; however, no statistical differences were observed when administered at eight weeks, supporting the concept that the use of melatonin is more effective during the early stages of bone healing.

The use of melatonin was also studied to enhance osseointegration in immediate implants placed in a dog mandible [45], obtaining a significant improvement of total BIC and inter-thread bone at 12 weeks. These findings agree with those obtained in implants placed in rabbit tibia, where the presence of well-remodelled cortical bone and new fronts of osteoblasts could be observed in animals treated with melatonin compared to the control group [44,46], as well as increased trabecular, but not cortical BIC. The effect of melatonin in zirconia implants was studied by the group of Calvo-Guirado [47], obtaining better results at one and four weeks in the treated implants.

Calvo-Guirado et al. [48] studied the effects of the application of melatonin or apigenin on post-extraction sockets in dogs. They evaluated the new bone area at 30, 60, and 90 days. The authors found that new bone percentage at 30 and 60 days was statistically significantly increased in the animals treated with melatonin, concluding that this molecule seems to stimulate the production of a greater number of cortical cells and accelerated considerably the synthesis and mineralisation of the osteoid matrix. In a previous study, it has been demonstrated that, in the immediate postoperative period following tooth extraction, there was an increase of the oxidative stress in the tissues, which can be counteracted by melatonin administration [49].

There is another study on the response of bone ingrowth around implants using melatonin and fibroblast growth factor-2 (FGF-2) in rats, where the use of a combination of both substances showed significant results in BIC with respect to the use of each of them separately and with the non-treated control group [40].

After implant placement, bone necrosis often occurs to a certain degree, and there is always an inflammatory reaction, as a direct consequence of surgery [50]. During and after the surgery, blood cells, such as macrophages and leukocytes, migrate to the peri-implant site and promote an increase in free-radical production [51,52]; melatonin can act as anti-inflammatory and antioxidant and may attenuate this normal reaction to surgery [49,53]. The anti-inflammatory properties of melatonin were studied in comparison with the indomethacin, a nonsteroidal anti-inflammatory drug, in a rat paw oedema model [54], with no differences being found; in this study, authors suggested that melatonin may act as a natural inhibitor of the cyclooxygenase functions, modulating the inflammatory activity of this enzyme.

### 3.3. Melatonin and Periodontal Disease

Periodontal disease is an inflammatory disorder characterised by gingival bleeding, periodontal pocket formation, and destruction of connective tissue attachment. This disease starts in the dental biofilm, with the stimulation of the immune response against bacteria. The most common form of periodontal disease in humans is plaque-induced gingival inflammation and may progress to more aggressive forms of periodontitis. In the advanced form of the disease, there is extreme loss of gingival tissue and alveolar bone.

An important aspect in periodontal disease is the generation of free radicals, some of which were derived from oral bacteria and others originating from the inflammation and the induced immune response [55,56]; the activation of the pro-inflammatory molecules results in the destruction of periodontal tissues. It has been suggested that an increase in both oxygen and nitrogen reactive species is responsible for the tissue oxidative damage in periodontitis [57]. This increase in free radicals co-exists with a decrease in the antioxidant defence; this imbalance may lead to a substantial deterioration of the periodontal tissues [58]. Melatonin plays an important role in the control of this disease due to its antioxidant and free-radical scavenging properties.

The potential therapeutic effects of melatonin in periodontitis have been documented in vitro, as well as in animal studies and clinical trials [59].

The relationship between periodontal status and the melatonin level in saliva is still inconclusive [60]. In a study measuring the relationship between the salivary melatonin and the degree of periodontal disease in humans, Cutando et al. [61] found that there was an inverse correlation between them; as the severity of the periodontal disease increases, the salivary melatonin level decreases, indicating that melatonin may act as a protector from bacterial infections. Similar findings were reported in another study in which authors compared the salivary and the gingival crevicular fluid levels of melatonin in four groups of patients with different grades of periodontal disease, finding that the more severe was the periodontitis, the lower melatonin the levels found, with significant differences between the healthy group and the two groups affected by the disease (chronic and aggressive periodontitis) [62]. This study also reported that the melatonin levels in saliva and gingival crevicular fluid were similar (with no significant differences), confirming the results obtained in previous studies [62]. In the study carried out by Gómez-Moreno [63], the authors found that patients with periodontal disease had a significantly lower plasma and salivary level of melatonin, maintaining a similar salivary/plasma melatonin ratio to that of the healthy subjects.

Of all the evaluated studies on melatonin levels in patients with periodontal disease, only one, conducted on diabetic people, found that with the worst periodontal status, the salivary melatonin levels were increased [64]. The authors explained this as a consequence of the oral inflammatory mediators. In the remaining studies, the correlation between salivary melatonin levels and periodontal disease was negative [61,62,63].

These low concentrations of melatonin found in saliva of patients with periodontal disease may be a result of its higher use as a free-radical scavenger and anti-inflammatory, because of the elevated level of reactive oxygen species and inflammation found in these patients.

In a study on ligature-induced periodontitis in rats, the treatment of the animals with melatonin seemed to alleviate gingival inflammation due to the inhibition of the production of inflammatory cytokines [65], and the authors concluded that melatonin could decrease the oxidative stress and periodontal inflammation through downregulation of inflammatory cytokines and restoring the concentration of antioxidants in the tissues.

Several research studies support the idea that melatonin could be used as a biomarker for monitoring the severity of periodontal disease [66,67], as well as a possible treatment strategy.

### 3.4. Other Effects of Melatonin on the Oral Cavity

Melatonin was used for vertical bone augmentation in a rat calvaria model [68]. Bone augmentation is important to regenerate bone in localised defects, where there is insufficient volume for dental implant placement, and the vertical bone augmentation is the most difficult to achieve, because it means producing bone in an area where it had not existed before [69]. In the study carried out by Shino et al., the new bone regeneration in a secluded space in rat calvaria was significantly greater in animals treated with melatonin than in those without it at 12 weeks, with a significant increase in the number of new blood vessels and osteoblast-like cells [68].

Another studied effect of melatonin refers to tooth development. Melatonin has a role in it by regulating cellular processes in odontogenic cells [70]. The receptor 1a for melatonin was expressed in secretory ameloblasts, in the cells of the stratum intermedium and stellate reticulum, in the external epithelial cells, in odontoblasts, and in dental sac cells in the tooth germs of humans [71], suggesting that this substance may have an effect on these structures for tooth development.

Melatonin has an oncostatic activity due to its anti-proliferative action, the stimulation of immunity, and the modulation of oncogene expression; its anti-inflammatory, antioxidant, and anti-angiogenic properties are also important [72]. The anticancer activity was proven in vitro and in preliminary studies in vivo [72], but the concrete mechanism by which melatonin suppresses cancer growth is still to be completely determined, several mechanisms being proposed, such as the repression of the hypothalamic-pituitary-reproductive axis, enhancement of immune function, and direct anti-proliferative effects through various receptors, including melatonin receptor 1a [73]. In addition to its proper anti-cancer activity, melatonin can be useful in cancer patients as a palliative therapy to reduce or control the side effects of chemotherapy, due to its anti-cachectic, anti-asthemic, and thrombopoietic properties [74], as well as its ability to protect oral tissues from ionising radiation, limiting the molecular damage to mitochondria and the presentation of oral mucositis [75].

The effects of melatonin in cancer were due to its ability to eliminate reactive oxygen species, which are messengers for cancer cell division [72] and the amplification of the antitumor activity of IL-2 [76]. With regard to oral cancer, it has been speculated that the restoration of melatonin receptor 1a expression, in an exogenous way, inhibits the growth of oral squamous cell carcinoma [77].

Finally, melatonin could be used in the oral cavity as a treatment for bacterial and viral infections. The beneficial effects of melatonin as an antiviral was studied in several types of infections (Venezuelan equine encephalomyelitis, West Nile encephalitis, etc.), where the use of melatonin as a treatment had beneficial effects in diminishing the viremia and the consequent mortality [78]. Regarding the diseases of the oral cavity, melatonin was studied in herpes virus treatment and was compared with the effect of acyclovir (a common treatment used for this disease) [79]. The results showed that the efficacy of melatonin in diminishing the severity of the infection was almost as effective as the antiviral drug, with the benefit of having fewer side effects. The benefit of melatonin in viral infections seems to be due to the immunomodulatory action in the stimulation of IL-1β, which has antiviral effects, as well as anti-oxidative and anti-inflammatory effects. Another role of melatonin in viral infections could be that of improving an immune system weakened by free radicals [80].

In bacterial infections, melatonin was used as a successful therapy in several in vivo models [81,82], with action against Gram-positive and Gram-negative microorganisms, but with a higher efficacy on the latter; it also showed efficacy against different strains of antibiotic-resistant bacteria [83]. The possible mechanism of action of melatonin as an antibiotic included the regulation of the proliferation/duplication of bacteria, the reduction of lipid uptake [83], and high metal binding capacity, including iron [84]. In the oral cavity, the antibacterial activity of melatonin could be useful in controlling periodontal disease and in dental caries induction [85].

## 4. Discussion

At pharmacological doses, melatonin caused the inhibition of bone resorption and an increase in bone mass, accelerating the mineralisation process of the bone matrix [28]. This could explain the greater volume of mineralised bone, as well as new bone formation around the implants treated with melatonin, as seen in all of the studies evaluated.

The use of melatonin in implants enhances an earlier osseointegration in different animal models [2,40,41,42,43,44,45,46,47]. Melatonin produces higher values of BIC and inter-thread bone, which is based on the direct action of this molecule on the osteoblasts, with a higher rate of maturation and of bone matrix production and mineralisation [45].

Melatonin appears to have a positive effect in the osseointegration of dental implants during the early stages of healing and could be used as a biomimetic agent during the implant surgery [2,86], making the healing process more effective, enhancing the initial conditions of the receptor tissues, reducing the time for osseointegration, and improving the patient’s quality of life. In most of the evaluated studies, the effect of melatonin was enhanced in the early phase after the implant placement, ranging from two to eight weeks, and the evidence of the melatonin benefits are less evident at later stages of the healing process [2,41,42,43,44]. It was observed that the circulating half-life of melatonin is approximately 23 min [87], thus, some authors speculate on the potential use of carriers in melatonin treatment in order to release it gradually and increase the half-life in the tissues, the increase of the melatonin effect during implant placement being particularly interesting [43].

Periodontal diseases are the result of the effects of bacteria and their products on the host response and it has been suggested that there are biological mediators, such as melatonin, which can contribute to the protection of periodontal tissues [88]. When suffering from periodontal disease, the amount of melatonin in saliva and in gingival crevicular fluid seems to be lower when the pathology is more severe, indicating that it may play a protective role against this disease, although further research is required to confirm this hypothesis. The protective role of melatonin on periodontal tissues might be explained to some extent by its antimicrobial action [60], its activation of the immune system [89], and its anti-inflammatory and free radical scavenger effect [90]. In addition, melatonin may modulate periodontal destruction by inhibiting prostaglandin E_2_ synthesis and, therefore, inhibit osteoclast differentiation [91], neutralising reactive oxygen species at the level of osteoclast lacuna, with the inhibition of bone resorption and stimulating osteoblast differentiation [30,63].

In cancer treatment, melatonin is used as a possible therapeutic target, with consistent effects against solid tumours, as well as a palliative therapy to reduce or control the side effects of chemotherapy. The different observations suggest that the use of melatonin should be considered in clinical trials, administered as a single treatment or in combination with other anticancer drugs [92].

## 5. Conclusions

In conclusion, the potential use of melatonin in oral diseases, such as implant placement or periodontitis, was studied by several groups, most of them with favourable results. Currently, there is no consensus for the best route of administration of this molecule, as well as in terms of the dosage needed for a good effect; thus, further research should be carried out in this sense. However, the relationship between melatonin levels in saliva and crevicular fluid and periodontal disease is not completely understood, and further studies should be conducted since the results were different depending on the evaluated study. Finally, clinical studies should also be performed, to observe the effects of melatonin on implant placements in humans.

## Figures and Tables

**Table 1:** Summary of studies focusing on the use of melatonin in dental implants placement.

Study	Animal Model	Animals/Implants	Time	Alone or Combination	Results
Cutando et al., 2008 [2]	Beagle dog	12/72	2 weeks	Alone	↑ BIC in melatonin group
Takechi et al., 2008 [40]	Wistar rat	10/20	4 weeks	+ FGF-2	↑ BIC and bone density in combination group
Calvo-Guirado et al., 2010 [41]	Beagle dog	12/48	4 weeks	+ porcine bone	↑ BIC, bone density and new bone in combination group
Guardia et al., 2015 [42]	Beagle dog	12/72	5 and 8 weeks	Alone	↑ Bone formation in melatonin group
Muñoz et al., 2012 [43]	Beagle dog	12/48	2, 5 and 8 weeks	+ Growth hormone	↑ BIC ando bone density at 2 and 5 weeks in combination group
Tresguerres et al., 2012 [44]	Rabbit	10/40	4 weeks	Alone	↑ trabecular BIC in melatonin group
Salomó-Coll et al., 2015 [45]	Foxhound dog	6/24	12 weeks	Alone	↑ total BIC in melatonin group
Calvo-Guirado et al., 2015 [47]	Rabbit	20/80	1 and 10 weeks	Alone	↑ BIC in titanium and zirconium implants at 1 week, in zirconium at 10 weeks
